# Long-acting Paliperidone in the Management of Severe Behavioral Disturbances Post-Traumatic Brain Injury: A Case Report

**DOI:** 10.1192/j.eurpsy.2025.1095

**Published:** 2025-08-26

**Authors:** A. Y. Al Ghailani, H. Mirza, S. Al Adawi

**Affiliations:** 1 Psychiatry, Oman Medical Speciality Board; 2 Behavioural Medicine, Sultan Qaboos University Hospital, Muscat, Oman

## Abstract

**Introduction:**

Traumatic brain injury (TBI) is a critical public health issue that often results in lasting cognitive and behavioral impairments.

These impairments include heightened risks for psychiatric disorders, such as aggression, social withdrawal, and mood instability.

Managing TBI-related behavioral symptoms can be complex, particularly in adolescent patients, where non-adherence to medication presents additional challenges.

**Objectives:**

To evaluate the effectiveness of long-acting injectable paliperidone palmitate in managing severe behavioral disturbances following TBI in an adolescent patient with a history of non-adherence to oral antipsychotic treatments.

**Methods:**

A case study approach was employed to detail the treatment and outcomes of a 16-year-old male who developed persistent aggressive and impulsive behaviors following a severe TBI at age 9.

The patient’s treatment history involved multiple oral antipsychotic trials, each limited by adherence issues or side effects, before transitioning to paliperidone palmitate injections.

Behavioral outcomes were monitored, and family-reported assessments of improvement were collected.

**Results:**

Following the initiation of monthly paliperidone palmitate injections at an initial dose of 100 mg, later increased to 150 mg, the patient showed significant reductions in aggression and improved behavioral control.

Family members observed fewer aggressive outbursts and better social interactions.

Mild side effects, including weight gain and an increase in prolactin levels, were reported, but these were generally well tolerated by the patient.

**Conclusions:**

This case demonstrates the potential benefits of long-acting injectable antipsychotics for managing behavioral dysregulation in TBI patients who struggle with medication adherence.

Paliperidone palmitate was effective in stabilizing the patient’s behavior, underscoring the need for tailored, multidisciplinary treatment plans that incorporate both pharmacological and psychosocial interventions to optimize long-term recovery in TBI-related neuropsychiatric care.

**Disclosure of Interest:**

None Declared

